# Shaping inhibition: activity dependent structural plasticity of GABAergic synapses

**DOI:** 10.3389/fncel.2014.00327

**Published:** 2014-10-27

**Authors:** Carmen E. Flores, Pablo Méndez

**Affiliations:** Department of Basic Neuroscience, Geneva Medical Center, University of GenevaGeneva, Switzerland

**Keywords:** GABAergic synapses, structural plasticity, activity-dependent plasticity, interneuron, memory, cerebral cortex, hippocampus

## Abstract

Inhibitory transmission through the neurotransmitter γ-aminobutyric acid (GABA) shapes network activity in the mammalian cerebral cortex by filtering synaptic incoming information and dictating the activity of principal cells. The incredibly diverse population of cortical neurons that use GABA as neurotransmitter shows an equally diverse range of mechanisms that regulate changes in the strength of GABAergic synaptic transmission and allow them to dynamically follow and command the activity of neuronal ensembles. Similarly to glutamatergic synaptic transmission, activity-dependent functional changes in inhibitory neurotransmission are accompanied by alterations in GABAergic synapse structure that range from morphological reorganization of postsynaptic density to de novo formation and elimination of inhibitory contacts. Here we review several aspects of structural plasticity of inhibitory synapses, including its induction by different forms of neuronal activity, behavioral and sensory experience and the molecular mechanisms and signaling pathways involved. We discuss the functional consequences of GABAergic synapse structural plasticity for information processing and memory formation in view of the heterogenous nature of the structural plasticity phenomena affecting inhibitory synapses impinging on somatic and dendritic compartments of cortical and hippocampal neurons.

## Introduction: GABAergic system as a substrate for brain plasticity

During the last decades neuroscientists and physiologists worldwide have made an herculean effort to elucidate the mechanisms of activity-driven changes in synaptic strength and understand its physiological significance as the brain substrate for learning and memory (Malenka and Bear, 2004; Mayford et al., 2012). Recent observations have added an additional level of complexity to activity-dependent synaptic plasticity of excitatory synaptic transmission by showing that adaptive changes in glutamatergic synapse strength are accompanied by a dynamic regulation of excitatory synapse structure (Yuste and Bonhoeffer, 2001; Matsuzaki et al., 2004). Activity-driven structural changes include actin-dependent enlargement of the postsynaptic density (Matus, 2000; Honkura et al., 2008) and formation and elimination of excitatory synapses (Holtmaat and Svoboda, 2009). *In vivo* studies have shown that dendritic spines, the morphological correlate of glutamatergic synapses in excitatory neurons, are formed and eliminated in response to synaptic activity patterns induced by learning behavior (Xu et al., 2009) and that formation of durable memories is directly correlated with the stability and formation of new excitatory synapses (Yang et al., 2009). In addition to functional plasticity, the structural rearrangement of glutamatergic synapses is critically involved in the brain processes leading to learning and memory formation (Caroni et al., 2012).

The plasticity of inhibitory neurotransmission has received relatively less attention than its excitatory counterpart despite its potential to deeply alter the function of cortical networks. A recent attempt to unravel different forms of plasticity in inhibitory γ-aminobutiric acid (GABA) releasing neurons has proven to be unexpectedly successful and cover many different aspects of the physiological properties of inhibitory cell’s, including glutamatergic inputs, dendritic and axonal structure, passive properties and GABAergic synapses onto target cells (Kullmann et al., 2012). As glutamatergic contacts, inhibitory synapses have the remarkable property of being able to alter the efficiency of synaptic transmission according to the patterns of activity that flow through them. Research carried out during the last two decades has made clear that inhibitory synapses undergo short- and long-term forms of plasticity and numerous examples of activity dependent changes in synaptic strength of GABAergic synapses have been described in different brain areas, including hippocampus and cortex (Gaiarsa et al., 2002; Castillo et al., 2011; Méndez and Bacci, 2011). The complex and varied collection of pre- and postsynaptic mechanisms that underlie the induction and expression of GABAergic synapse plasticity mirrors the heterogeneous nature of different inhibitory neuron subtypes that form cortical GABAergic synapses (Méndez and Bacci, 2011).

**Figure 1:**
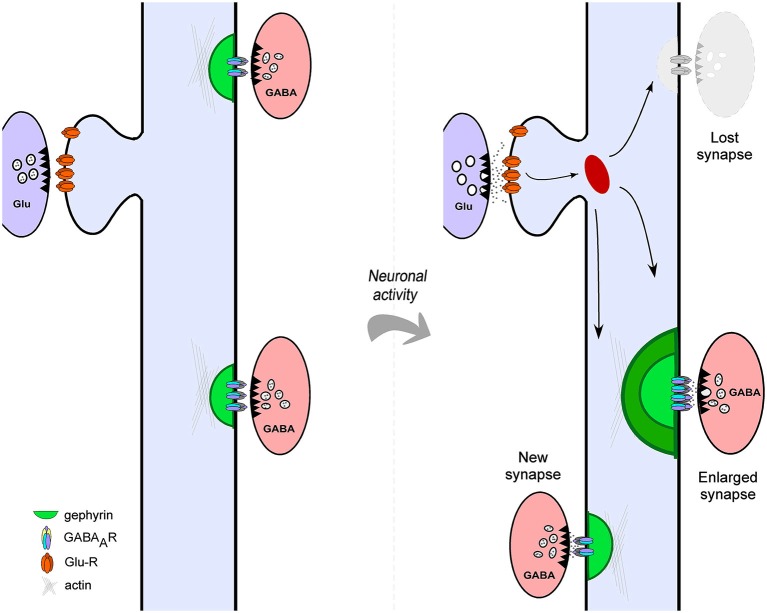
**Structural plasticity of cortical GABAergic Synapses**. Neuronal activity drives different forms of structural plasticity of gephyrin-containing inhibitory synapses. Neuronal activity may alter the size of pre-existing inhibitory contacts (left panel) or trigger complete elimination of GABAergic synapses (shaded gray, right panel). In addition, new gephyrin clusters are formed at different dendritic locations in response to altered levels of network activity (right panel).

It is now clear that, similar to excitatory synapses, the molecular composition and structure of GABAergic synapses show a high degree of dynamism (Kittler and Moss, 2003; Lévi et al., 2008; Chen et al., 2012; van Versendaal et al., 2012). The changing nature of inhibitory synapse structure raises the possibility that functional alterations in inhibitory neurotransmission may occur through structural rearrangements. Indeed, synaptic activity driven functional changes of inhibitory neurotransmission are accompanied by modifications in the structure of GABAergic synapses with two major consequences: alteration of synaptic size and morphology and formation and elimination of inhibitory contacts (Figure 1). Here we review several aspects of the structural plasticity of mammalian cortical and hippocampal GABAergic synapses. What is the nature of the morphological changes affecting hippocampal and cortical inhibitory synapses? What is the role of synaptic activity and experience in the induction of structural alterations of inhibitory synapses and what molecular mechanisms underlie this form of plasticity? In view of the staggering diversity of different inhibitory neuron subtypes that configure cortical networks, what are the potential physiological consequences of the structural remodeling of GABAergic synapses? We attempt to answer these questions with the aim of providing a framework to understand the characteristics, mechanisms and functions of inhibitory synapse structural remodeling.

## GABAergic synapses and functional plasticity

**Figure 2:**
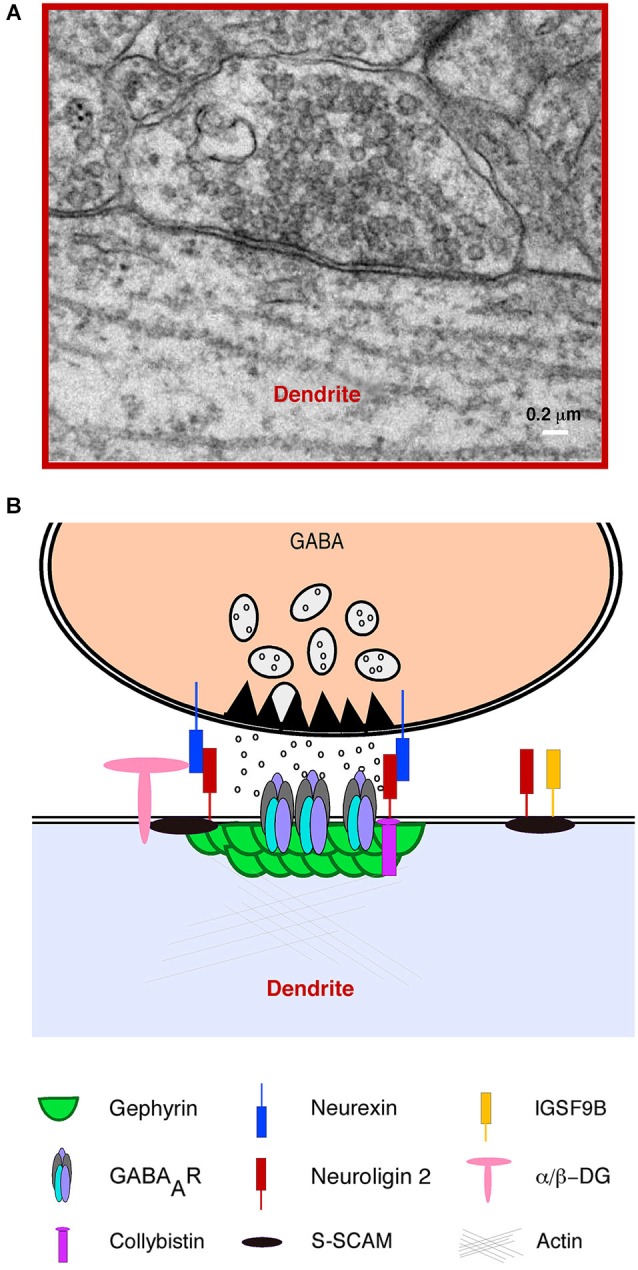
**Structural features and molecular composition of an hippocampal GABAergic Synapse. (A)** Electron microscopy (EM) image of an inhibitory (symmetrical synapse) between a GABAergic presynaptic terminal and a proximal apical dendrite of a CA1 hippocampal pyramidal neuron. The image shows typical ultrastructural features of inhibitory synapses: a distinguishable synaptic cleft, pleomorphic GABA containing vesicles and a thin post synaptic density (PSD) facing an active zone of similar width. **(B)** A simplified cartoon of a typical GABAergic synapse illustrating the presynaptic terminal with vesicles containing GABA, the active zone, presynaptic neurexins and the inhibitory postsynaptic density showing a vast number of postsynaptic proteins including GABA_A_ receptors and the scaffold protein gephyrin.

Inhibitory synapses, also known as symmetrical synapses (or type II) by their ultrastructural features (Figure 2A; Gray, 1959; Colonnier, 1968) are arranged around the scaffold protein gephyrin, the main molecular organizer of inhibitory synapses (Sassoè-Pognetto et al., 2011; Tyagarajan and Fritschy, 2014). Gephyrin forms submembranous hexagonal macromolecular complexes (Xiang et al., 2001; Fritschy et al., 2008) that orchestrate multiple protein-protein interactions with GABA_A_ Receptors (GABA_A_ R), the cytoskeleton and various cell adhesion and signal transduction proteins (Figure 2B).

Inhibitory synapses are non-uniformly distributed along the different subcellular compartments of pyramidal cells. In the hippocampus, the highest density of GABAergic inputs is found in the soma and proximal dendrites compared to intermediate and distal dendrites (Megías et al., 2001). GABAergic synapses are formed by a highly heterogeneous group of cells (Markram et al., 2004; Ascoli et al., 2008) that mostly correspond to the definition of interneuron (locally projecting neuron with axonal arborization, dendritic and somatic compartments in the same anatomic structure). Interestingly, some interneurons (INs) form synapses exclusively on the dendrites of other neurons (Maccaferri, 2005), while other target the somatic compartment (Freund and Katona, 2007). This highly stereotyped axonal targeting of GABAergic synapses to specific cellular compartments allows different INs subtypes to selectively affect the different computational processes that actively integrate synaptic inputs in the soma and dendrites (Miles et al., 1996; Pouille and Scanziani, 2001, 2004). Adult INs have a critical role in maintaining physiological activity levels, stabilizing neuronal networks and preventing runaway excitation through different forms of GABA mediated inhibition. In addition, INs are able to form synaptic contacts with a large number of neighboring neurons and provide synchronous inhibition to functionally significant portions of the network (McBain and Fisahn, 2001). In this way, INs coordinate the spiking activity of large number of cells and are critically involved in the genesis of the wide variety of rhythmic network activities that are the basis of cognition and behavior (Klausberger and Somogyi, 2008; Buzsáki and Wang, 2012).

### Functional plasticity of cortical GABAergic synapses

The heterogeneous population of cortical INs shows an equally diverse range of mechanisms of activity driven changes in synaptic strength of GABAergic neurotransmission. Retrograde signaling has been shown to play a prominent role in the modulation of GABAergic synaptic plasticity. Activity-dependent synthesis and release of different signaling molecules by post-synaptic excitatory neurons induce short and long-term forms of plasticity in cortical inhibitory synapses. Endocannabinoids are synthesized by postsynaptic neurons in response to increased Ca^2+^ concentrations, action potential trains and metabotropic glutamate, dopamine, and acetylcholine receptor activation (Kano et al., 2009). Endocannabinoids travel retrogradely and activate CB1 receptor, a G-protein coupled receptor located mainly on presynaptic terminals, resulting in short or long term decreases in GABA release (Piomelli, 2003; Chevaleyre et al., 2006). Another form of desinhibition by retrograde signaling is mediated by postsynaptic somatodendritic glutamate release and activation of presynaptic metabotropic glutamate receptors (Zilberter, 2000). In addition, several examples of spike-timing dependent plasticity of GABAergic synapses have been described in the cortex and hippocampus. This form of plasticity requires near coincident pre- and postsynaptic spiking. The precise rules that dictate the sign of plasticity, potentiation or depression of GABAergic synapses seem to differ between hippocampus (Woodin et al., 2003), entorhinal cortex (Haas et al., 2006) and neocortex (Holmgren and Zilberter, 2001). High frequency stimulation of synaptic inputs has also the potential to produce long term depression (LTD) and potentiation of GABAergic neurotransmission (Komatsu, 1996; Komatsu and Yoshimura, 2000; Patenaude et al., 2003) by pre- (Chevaleyre and Castillo, 2003) and postsynaptic mechanisms (Lu et al., 2000). Astrocyte-dependent Ca^2+^ signaling (Kang et al., 1998) has been shown to modulate the strength of GABA synaptic signaling in the hippocampus. In the neocortex layer 4 excitatory neurons coupling of postsynaptic subthreshold depolarizations with presynaptic action potentials of presynaptic INs triggers a form of long term potentiation (LTP) of GABAergic synaptic transmission that is modulated by sensory activity (Maffei et al., 2006).

All these forms of induction and expression of functional plasticity at inhibitory GABAergic synapses occur through a large variety of mechanisms. Changes in GABAergic synaptic strength are mediated by mechanisms that range from post-transcriptional modifications of GABA receptors such as phosphorylation (Vithlani and Moss, 2009), ubiquitination (Saliba et al., 2007), trafficking (Vithlani et al., 2011) and lateral diffusion (Lévi et al., 2008; Bannai et al., 2009), to alterations in presynaptic GABA release and variations in chloride (the main GABA_A_ receptor permeable ion) driving force (Woodin et al., 2003) and give rise to short and long terms changes in inhibitory synapse efficacy. Other forms of plasticity of inhibitory transmission depend on GABA and glutamate postsynaptic signaling through metabotropic and ionotropic receptors (Lu et al., 2000; Wang and Maffei, 2014). Although during the last decades our knowledge of the modes and mechanisms of activity dependent changes in GABAergic synaptic strength has grown enormously (Kullmann et al., 2012), we have a limited knowledge of the structural remodeling that accompanies most of these functional changes. Structural remodeling of GABAergic synapses may represent an essential mechanism for activity dependent regulation of GABAergic function.

## Structural plasticity of GABAergic synapses

Examples of activity mediated structural plasticity of GABAergic synapses have been observed during the development of the mammalian brain. Brain patterning occurs through genetic programs that guide the generation and migration of INs and the innervation patterns, geometry and target specificity of GABAergic axonal projections (Hébert and Fishell, 2008; Bartolini et al., 2013). In mice, maturation of GABAergic connectivity occurs both embryonically and during the first postnatal weeks, leaving open the possibility of an experience dependent modulation of inhibitory synapse formation. Indeed, during early stages of brain formation, experience and activity dependent processes overlap with genetically encoded mechanisms of development and regulate several aspects of GABAergic synaptogenesis, including axonal branching, formation of GABAergic synaptic contacts and synaptic strength (Huang et al., 1999; Doischer et al., 2008; Huang, 2009). It has been shown that reduced synaptic activity induced by sensory deprivation in young, but not adult animals, produces specific reductions in the number of perisomatic inhibitory synapses on cortical excitatory cells, unmasking a regulatory role of neuronal activity in determining the density of inhibitory cell contacts in this specific cellular compartment (Jiao et al., 2006). Other studies have provided similar results using pharmacological and genetic manipulation of activity in developing hippocampal cultures (Marty et al., 2000; Hartman et al., 2006). Interestingly, these studies point to a homeostatic role of inhibitory synapse structural plasticity in compensating alterations in global activity levels of developing cortical and hippocampal networks. GABA content in INs is a critical mediator of GABAergic innervation in the developing visual cortex. Knocking down Glutamic Acid Decarboxilase 67 (GAD67), the main GABA synthesizing enzyme in cortical INs, resulted in serious deficits in axonal branching and decreased formation and size of perisomatic synapses on cortical pyramidal neurons (Chattopadhyaya et al., 2004, 2007). The regulation of GAD67 expression and function by synaptic activity suggests that GABA itself could be a mediator of activity dependent structural remodeling of GABAergic synapses.

### Activity dependent ultrastructural changes in GABAergic synapses

Subtle activity induced changes in adult GABA synapse morphology have been studied using electron microscopy (EM), which allows the unequivocal identification of symmetrical GABAergic synapses and the analysis of synaptic ultrastructure at very high resolution. This technique has shown that patterns of activity that produce functional and structural changes in excitatory synapses also induce structural remodeling of inhibitory synapses. Both *in vitro* (Lushnikova et al., 2011) and *in vivo* (Nusser et al., 1998), the rise in synaptic activity levels increased inhibitory synaptic junctional area and complexity and proportion of somatic cell surface covered with inhibitory postsynaptic densitiy (PSD). In other cases, different aspects of inhibitory synapse plasticity occur in a coordinated manner. Bourne and Harris (2011) used LTP inducing protocols and EM three dimensional reconstructions of CA1 pyramidal neurons dendritic segments in acute hippocampal slices to show that plasticity inducing protocols produce a decrease in dendritic inhibitory PSD density that is counterbalanced by an extension of the individual PSD areas. These experiments demonstrate that the adult inhibitory PSD is endowed with mechanisms that allow dynamic changes in the structure in response to alterations in the levels of network activity.

Chronic sensory deprivation by whisker trimming induces a net decrease in the number of symmetric GABAergic synapses in the dendrites of layer 4 neurons of barrel cortex, the main target neurons for the thalamocortical axons relaying sensory information from the whiskers (Micheva and Beaulieu, 1995). Artificially increasing single whisker activity by passive stimulation leads to a rise in dendritic inhibitory synapse density in the correspondent barrel but not in the neighboring ones (Knott et al., 2002). Interestingly, sensory deprivation (through whisker trimming) and stimulation (by artificial chronic movement of the whiskers) result in opposite effects on the number of dendritic inhibitory synapses by preferentially affecting inhibitory synapses contacting dendritic spines and, to a much lower extent, those formed in the shaft of dendrites of principal cells (Micheva and Beaulieu, 1995; Knott et al., 2002). Inhibitory synapses of the barrel cortex are also remodeled in response to learning. A conditioning paradigm involving a group of whiskers induces an increase in the number of symmetric synapses and GABA content of inhibitory axons impinging on dendrites of layer 4 pyramids of the barrel corresponding to the trained whiskers (Jasinska et al., 2010). As in the cases described above, this de novo, learning induced GABAergic synaptogenesis affected inhibitory contacts on dendritic spines but not those on the dendritic shafts of layer 4 excitatory neurons. It is clear that a direct positive relation exists between synaptic activity and GABA synapse formation in the adult somatosensory cortex and that spine GABA synapses, represent a highly structurally dynamic pool of synapses.

### Inhibitory axon plasticity

A major difficulty in the study of inhibitory synapse dynamics is the lack of morphological markers at the optical level. However, different approaches have been used to track cortical GABAergic structural plasticity. Transgenic mouse lines with genetically labeled subpopulation of INs allow the visualization of inhibitory axon dynamics in organotypic hippocampal slice cultures and in the cortex *in vivo*. A pioneer study gained insight in the mechanisms of formation of new inhibitory synapses on CA1 pyramidal cell dendrites *in vitro* using high resolution fluorescence confocal imaging of the sites of contact of inhibitory axons and postsynaptic structures (Wierenga et al., 2008). Although the vast majority of putative contact sites were stable, the authors were able to detect formation of new inhibitory contacts between GABAergic axons and dendrites of excitatory neurons. Interestingly, new stable contacts were formed at location were pre- and postsynaptic structures were in close apposition (Wierenga et al., 2008). Close observation of the presynaptic component alone has shown that the majority of putative presynaptic structures is stable and shows abundant expression of inhibitory pre and post synaptic proteins (Schuemann et al., 2013). Short-lived boutons show however, lower levels of GABA synapse markers (Schuemann et al., 2013), suggesting a protracted maturation of new GABAergic contacts. Interestingly, the level of network activity directly controlls inhibitory axon plasticity and produces subtle but significant changes in bouton turnover and morphology (Schuemann et al., 2013). Tracking GABAergic axons dynamics *in vivo* has been possible by implanting cranial widows in mice expressing a fluorescent protein in a subpopulation of inhibitory neurons and visualizing inhibitory presynaptic structures using confocal microscopy in superficial cortical layers (Keck et al., 2011). A certain degree of inhibitory axons structural remodeling has been observed even in conditions of normal sensory activity in the somatosensory cortex: while the length of axonal projections from inhibitory cells in the barrel cortex remains constant, boutons are added and eliminated at a rate of 10% per week (Marik et al., 2010). However, under conditions of altered sensory input by whisker removal, axons from inhibitory neurons in the deprived barrels suffer intense structural modifications, retracting terminals in the vicinity of their cell body and extending collaterals beyond its normal projection range towards non-deprived barrels two days after sensory deprivation. In addition, whisker trimming produces at the same time a general decrease in bouton density (Marik et al., 2010). Similar manipulation of sensory inputs in the visual cortex, synaptic input deprivation by permanent lesion of the retina, induces the disappearance of a fraction of GABAergic boutons in few hours (Keck et al., 2011), suggesting that the rapid loss of functional inhibitory synapses may represent a general adaptive mechanism to decreased levels of synaptic activity that is conserved in different functional areas of the cortex. Axons of GABAergic neurons are dynamic structures, able to alter structural properties in response to altered levels of synaptic activity and sensory experience. By growing and retracting axons, INs are able to increase or decrease the number and change the identity of their postsynaptic targets. The appearance and elimination of boutons suggest the involvement of a mechanism that coordinates changes in pre- and postsynaptic structures during structural plasticity of GABAergic synapses.

### The postsynaptic side plasticity

**Figure 3:**
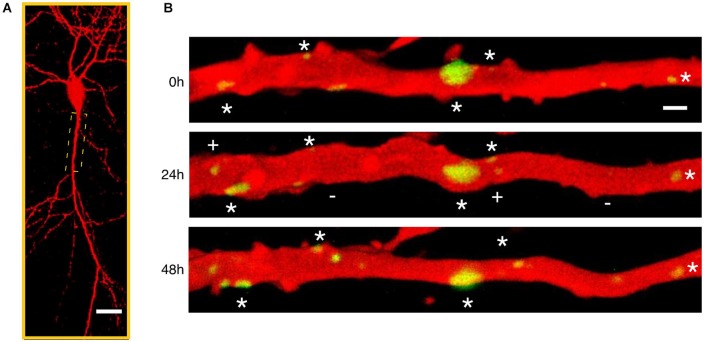
**Time-lapse imaging of gephyrin containing inhibitory synapses. (A)** Low magnification confocal microscope view of a CA1 pyramidal neuron in a mature hippocampal organotypic culture imaged after biolistic transfection with Red Fluorescent Protein to reveal neuronal structure and Green Fluorescent Protein tagged gephyrin to visualize the postsynaptic component of inhibitory synapses. **(B)** Repetitive laser scanning confocal imaging of an apical proximal dendrite (boxed region in **A**) taken every 24 h during 3 consecutive days. A large fraction of gephyrin-containing inhibitory synapses (green) are stable throughout the imaging period (stars) despite the high level of structural variability. In addition, gephyrin clusters appear (plus (+) sign) and disappear (minus (−) sign) at different dendritic locations suggesting continuous formation and elimination of inhibitory synapses.

Although tracking presynaptic structures identifies changes in putative inhibitory synaptic contacts the fluorescent tagging of the scaffolding protein gephyrin allows dynamic visualization of the postsynaptic component of GABAergic synapses (Figure 3). During developmental inhibitory synaptogenesis, gephyrin accumulates progressively at sites of new synapse formation following a similar pattern as presynaptic components such as the vesicular GABA transporter VGAT, (Dobie and Craig, 2011). Developing inhibitory postsynapses show a high degree of structural plasticity including translational movements along dendrites in a coordinated manner with presynaptic axons and trafficking of synaptic vesicles from pre-existing boutons to new ones (Dobie and Craig, 2011). However, synaptic activity drives the maturation of inhibitory neurotransmission and results in stable and functionally stronger GABAergic synapses compared with those observed during the early phases of inhibitory synaptogenesis (Dobie and Craig, 2011; Vlachos et al., 2013).

The use of postsynaptic tagging of inhibitory synapses has the advantage of resolving the subcellular compartments where GABAergic axons impinge on postsynaptic neurons. Using this approach, two recent studies (Chen et al., 2012; van Versendaal et al., 2012) have revealed important features of structural plasticity of GABAergic synapses in visual cortical circuits *in vivo*. Under normal conditions, a continuos turnover affects a low proportion of GABAergic synapses in dendrites of layer 2/3 pyramidal neurons of the visual cortex. New GABA synapses replace lost inhibitory contacts and tend to be persistent in the majority of the cases (Chen et al., 2012), which contrasts with the low stability of new excitatory connections observed in young and adult animals (Holtmaat et al., 2005). GABAergic synapses on apical dendrites of layer 2/3 pyramidal neurons are preferentially removed in response to decreased sensory inputs by visual deprivation. Interestingly, not all GABAergic synapses on dendrites of pyramidal cells seem to be equally plastic. The most dynamic population of GABA synapses are those contacting dendritic spines (specially abundant in distal parts of layer 2/3 cortical pyramidal neurons) compared with inhibitory synapses made on the dendritic shafts (Chen et al., 2012; van Versendaal et al., 2012). Inhibitory synapses are thus dynamic structures formed and eliminated in cortical neurons *in vivo* in response to physiological changes in network activity levels. Under these same conditions, no alteration in density or turnover of excitatory connections is observed in the same cells (Hofer et al., 2009), suggesting that structural plasticity of GABA synapses and subsequent rewiring of inhibitory microcircuits is a fundamental mechanism for brain adaptation to sensory experience in layer 2/3 pyramidal cells.

## Molecular mechanisms of activity-dependent structural plasticity of GABAergic synapses

The experiments discussed above clearly show that network activity is a major driving force for structural remodeling of inhibitory synapses. Central to the molecular machinery that links synaptic activity with such plastic changes is the Neuronal Per Arnt Sim domain protein 4 (NPAS4), a brain-specific basic helix-loop-helix transcription factor, whose expression is tightly regulated by synaptic activity and postsynaptic calcium influx (Flavell and Greenberg, 2008). Neuronal Per Arnt Sim domain protein 4 regulates the formation of somatic and dendritic inhibitory synapses during development with little effect on excitatory contacts (Lin et al., 2008). In addition, experience-dependent inhibitory synapse formation critically depends on NPAS4-mediated gene expression and results in differential modulation of somatic and dendritic inhibition (Bloodgood et al., 2013). Interestingly, NPAS4 is a critical mediator of activity dependent expression of the neurotrophin Brain Derived Neurotrophic Factor (BDNF) that regulates developmental and activity dependent formation and elimination of inhibitory synapses (Marty et al., 2000; Berghuis et al., 2004; Jovanovic et al., 2004; Kohara et al., 2007). Structural plasticity of GABAergic synapses is at least in part controled by a postsynaptic mechanism that links neuronal activity with alteration in nuclear gene expression.

In a pioneering attempt to elucidate the molecular mechanisms of inhibitory synaptic plasticity, Nusser and colleagues (Nusser et al., 1998) used a combination of quantal analysis of evoked inhibitory postsynaptic currents with quantitative immunogold labeling of synaptic GABA_A_ Rs in hippocampal granule cells. They found that an increase in amplitude of synaptic currents corresponded to a proportional increase in the number of GABA_A_Rs at inhibitory synapses on somata and axon initial segments of hippocampal granule cells. The increased density of GABA_A_ Rs was accompanied by an enlargement of synaptic area and presynaptic boutons (Nusser et al., 1998). Although our knowledge of the mechanisms of activity-dependent structural plasticity of inhibitory synapses is still limited, this study suggests that, like excitatory synapses, inhibitory synaptic plasticity might not only depend on changes in the biophysical properties of the channels, but might also involve a structural reorganization of its inhibitory postsynaptic density, by altering the number of channels and the necessary structural components. Recent research shows that gephyrin forms dynamic domains within the inhibitory PSD that change size, form and localization in minutes (Specht et al., 2013). In the the hexagonal bidimensional lattice formed by gephyrin at the postsynaptic membrane, every single gephyrin interacts with GABA receptors in a one to one ratio approximately (Specht et al., 2013). Increasing synaptic gephyrin would then create new slots for allocating inhibitory receptors. Interestingly, functional and structural plasticity of excitatory synapses share a complete set of molecular machinery that allows coordinated changes in synaptic strength and architecture (Lüscher et al., 2000). Increasing our knowledge of the molecular mechanisms that regulate clustering of gephyrin and trafficking of GABA_A_ R at inhibitory synapses will surely increase our understanding of molecular mechanisms that regulate structural plasticity of GABAergic synapses.

### Activity dependent regulation of Gephyrin function

One of the most intensively studied molecule involved in the inhibitory synapse function is gephyrin, the major scaffold protein of the inhibitory synapse. Crystallographic techniques show that gephyrin oligomerizes via G-domain trimerization and E-domain dimerization forming an hexagonal submembrane lattice (Xiang et al., 2001). Gephyrin undergoes several post-translational modifications that affect its clustering and the interactions with GABA_A_Rs and a variety of regulatory proteins, signaling complexes and cytoskeleton (Tyagarajan and Fritschy, 2014). Dejanovic et al. (2014) have recently identified gephyrin palmitoylation as a mechanism of gephyrin synaptic targeting. As for PSD95 in excitatory synapses, gephyrin palmitoylation is essential for the interaction with cell membrane and therefore facilitates its postsynaptic clustering at GABAergic synapses. Gephyrin palmitoylation is dependent of synaptic activity since the application of bicuculline that blocks GABA_A_ Rs and increases network activity reduces gephyrin palmitoylation. In contrast, GABA application increases gephyrin palmitoylation (Dejanovic et al., 2014). Additionally, synaptic activity may alter gephyrin clustering via modulation of nitric oxide (NO) synthesis by neuronal NO synthase (Dejanovic and Schwarz, 2014).

Phosphorylation is a critical regulator of gephyrin function (Moss et al., 1995; Zacchi et al., 2014). Some of the signaling cascades that modulate gephyrin phosphorylation and GABAergic structural plasticity could represent a cross-road between excitatory and inhibitory synapse function, thus providing a mechanism of extreme importance to maintain the homeostasis of synaptic networks. Clear examples for such common molecular players are Glycogen Synthase Kinase 3 (GSK3β) and extracellular signal-regulated protein kinase/mitogen-activated protein kinase (ERK). At excitatory synapses, GSK3β phosphorylates PSD95 and modulates excitatory synaptic plasticity in an N-Methyl-D-Aspartate (NMDA) receptor-dependent manner by inducing α-Amino-3-hydroxy- 5-Methyl-4-isoxazolepropionic acid (AMPA) receptor internalization and thus LTD (Nelson et al., 2013) while ERKs boost excitatory synaptic and structural plasticity at different levels (Zhu et al., 2002; Patterson et al., 2010). At inhibitory synapses, gephyrin phosphorylation by both GSK3β and ERK1 at at Ser 270 and Ser 268 respectively modulates the structure and function of GABAergic synapses by altering the number and size of gephyrin clusters (Tyagarajan et al., 2011b, 2013). Glycogen Synthase Kinase 3-dependent phosphorylation decreases gephyrin cluster size, whereas phosphorylation by ERK decreases size and density of postsynaptic gephyrin clusters and is critical for the Ca2+-dependent cysteine protease (calpain-1) degradation of gephyrin. Phosphorylation of gephyrin by GSK3β and ERK1 is accompanied by parallel decrease in GABAergic mIPSCs. ERK and GSK3β activity are tightly regulated by neuronal activity making gephyrin phosphorylation a key mechanism for the coordination of structural remodeling of GABAergic synapses and network activity levels.

### Ca2+/calmodulin dependent protein kinase II regulates GABAergic synapse function and structure

Trafficking and stability of GABA_A_ Rs can be modulated by direct phosphorylation of the channel subunits (Vithlani et al., 2011). Several kinases target GABA_A_ R such as Protein Kinase A (Brandon et al., 2003; Jovanovic et al., 2004), Protein Kinase C (Brandon et al., 2002) and Ca2+/calmodulin-dependent protein kinase II (CaMKII; McDonald and Moss, 1997). CaMKII directly phosphorylates α1, β2, β3, and γ2 subunits (McDonald and Moss, 1997; Churn et al., 2002; Houston et al., 2009; Petrini et al., 2014). Activation of the NMDA subtype of glutamate receptors has been linked to potentiation of GABA_A_ R-mediated currents in different brain regions through activation of CaMKII (Marsden et al., 2007; Petrini et al., 2014). In addition, CaMKII translocates from dendritic spines to inhibitory synapses upon weak chemical stimulation by NMDA and promotes the insertion of GABA_A_ Rs to inhibitory synapses and enhancement of inhibitory transmission (Marsden et al., 2010). Are these functional changes accompanied by structural remodeling? A recent published work by Petrini et al. (2014) found that the chemical inhibitory long term potentiation (iLTP) triggered by NMDA application (up to 30 min) requires CaMKII-dependent phosphorylation of GABA_A_ R subunit β3 at serine 383 that in turn promotes the synaptic recruitment of gephyrin from extrasynaptic sites. The increase of gephyrin at synapses is not explained by de novo synthesis, but rather by a regulation of the mechanisms that control the redistribution of gephyrin. In turn, using single-particle tracking of quantum dots labeled GABA_A_Rs, they found that gephyrin recruitment at the synapses stabilizes GABA_A_ Rs. The recruitment of gephyrin by an activity dependent phosphorylation of GABA_A_ R subunit represents a way in which functional changes are coordinated with structural changes.

### Cell adhesion molecules and activity dependent regulation of GABAergic synapse

Gephyrin clustering at GABAergic synapses requires its interaction with a neuron specific Guanine Nucleotide Exchange Factor (GEF) Collybistin (CB; Kins et al., 2000; Harvey et al., 2004; Papadopoulos et al., 2007). CB is expressed specifically in neurons and activates cell division control protein 42 homologue (Cdc42; Xiang et al., 2006) and the small Rho-like GTPase TC10 (Mayer et al., 2013) that in turn regulate cluster formation and the aggregation GABA_A_ Rs (Poulopoulos et al., 2009; Tyagarajan et al., 2011a). Inhibitory synapse formation, maturation, maintenance and function are also regulated by synaptic cell adhesion molecules (CAMs; Yamagata et al., 2003; Gerrow and El-Husseini, 2006). At inhibitory synapses, one of the most prominent CAMs belongs to the Neuroligin family (Craig and Kang, 2007; Südhof, 2008). Neuroligin 2 (NL2) is specifically localized at inhibitory synapses (Varoqueaux et al., 2004) where it modulates their formation, maturation and function (Graf et al., 2004; Chih et al., 2005; Chubykin et al., 2007). NL2 deficient mice lack postsynaptic specialization at perisomatic inhibitory synapses (Poulopoulos et al., 2009) and show decreased inhibitory synaptic transmission (Chubykin et al., 2007). Through a conserved cytoplasmatic domain, NL2 binds gephyrin (Poulopoulos et al., 2009) while an extracelullar motif mediates NL2 trans-synaptic interaction with presynaptic Neurexins (NRXs) boosting inhibitory presynaptic axonal differentiation during development (Chih et al., 2005). The interaction with the scaffold protein gephyrin brings NL2 close to GABA receptors and other gephyrin binding proteins (Fritschy et al., 2012). NRXs are CAMs that have two isoforms, α-NRXs and β-NRXs (Südhof, 2008). α-NRXs are expressed primarily at GABAergic synapses, whereas β-NRXs are localized at both excitatory and inhibitory synapses (Chih et al., 2005) where they form a dense transynaptic assembly (Tanaka et al., 2012). Interestingly, β-NRX1 has high turn over rate in presynaptic membranes and is stabilized by neuronal activity and GABA release (Fu and Huang, 2010). In addition, NRXs directly interact with GABA_A_ Rs, thus modulating GABAergic transmission in a NL independent fashion (Zhang et al., 2010).

GABA dependent stabilization of presynaptic NRXs may represent a mechanism for activity dependent GABAergic synapse remodeling. NRXs stabilized by local GABA release (Fu and Huang, 2010) drive the clustering of NL2 postsynaptically. Neuroligin 2 interaction with gephyrin brings CB close to NL2, where CB/NL2 interaction releases SH3 domain and activates CB (Poulopoulos et al., 2009). Interaction with GABA_A_R α2 subunit may also activate CB (Tretter et al., 2008). Activated CB leads gephyrin and GABA_A_ Rs to the postsynaptic membrane and further stabilizes them at the synapses. Neuroligin 2 initiates clustering of other molecules essential for synapse function (Lévi et al., 2002; Woo et al., 2013; Pribiag et al., 2014) strengthening in this way synaptic adhesion. Finally, NL2 intracellular interactions with γ2 subunit of GABA_A_R stabilize both molecules at inhibitory synapses (Dong et al., 2007). Thus, GABA unchained downstream signaling may play a role in coordinating the formation and remodeling of the pre- and postsynapses (Fritschy et al., 2008; Tretter et al., 2012).

## Functional role and specificity

The study of structural plasticity of glutamatergic synapses has focused on the relationship between morphological parameters of dendritic spines and physiological properties of its excitatory synapse (Nimchinsky et al., 2002; Bourne and Harris, 2008; Kasai et al., 2010). Recent studies have shown that although inhibitory post-synapses do not have such morphological fingerprint, fluorescently tagged gephyrin can be used to visualize inhibitory synapses and track dynamic changes (Dobie and Craig, 2011; Chen et al., 2012; van Versendaal et al., 2012). Indeed, virtually all gephyrin clusters detected at the optical level using fluorescent microscopy had a correlate with a GABAergic synapse detected at the ultrastructural level using EM (Chen et al., 2012; van Versendaal et al., 2012). Although the functional correlation between optically measurable parameters such as size and intensity of gephyrin clusters and inhibitory synapse function is currently unknown, EM studies have shown that increased synaptic strength in inhibitory synapses produces a coordinated insertion of GABA_A_Rs and enlargement of the PSD (Nusser et al., 1998). Whether such structural rearrangement can be visualized at the optical level using fluorescently-tagged gephyrin or other inhibitory synapse fluorescent markers requires further research that will provide invaluable information about structure-function relationship in inhibitory synapses.

During brain development, when structural dynamism of GABAergic synapses is high, the functional consequences of inhibitory synaptic rearrangements are determined by the depolarizing effects that activation of GABA_A_Rs has on postsynaptic target cells (Cherubini et al., 1991). In adulthood, however, coordination of the amount of excitatory and inhibitory inputs becomes an essential function of structural plasticity of GABAergic synapses. Both perisomatic (Nusser et al., 1998; Lushnikova et al., 2011) and dendritic inhibitory synapses (Knott et al., 2006; Jasinska et al., 2010; Chen et al., 2012; van Versendaal et al., 2012) show high levels of structural plasticity, suggesting that excitation/inhibition balance could be controled independently in different subcellular compartments. Indeed, recent reports show that active excitatory synaptic inputs are not randomly distributed along dendritic arbors in principal cells neurons (Kleindienst et al., 2011; Makino and Malinow, 2011; Takahashi et al., 2012). The ability of inhibitory synapses to target specific subcellular compartments could allow local control of such clustered inputs. In addition, an homeostatic role is a reasonable interpretation for studies showing simultaneous rearrangement of perisomatic inhibitory and excitatory synapses in response to patterns of activity known to induce potentiation of glutamatergic synaptic transmission (Nusser et al., 1998; Bourne and Harris, 2011; Lushnikova et al., 2011). In the barrel cortex, increases and decreases in whisker-induced neuronal activity are directly related to the formation or elimination of GABAergic spine synapses respectively (Micheva and Beaulieu, 1995; Knott et al., 2002). This form of GABAergic structural plasticity may participate in the homeostatic adjustment of circuit activity levels after long-term changes in somatosensory evoked synaptic activity. It is increasingly clear that patterns of synaptic activity that induce structural rearrangements of inhibitory circuits also induce plasticity (functional and/or structural) of excitatory synapses. Functional plasticity of GABAergic synapses has been shown to modulate excitatory neurotransmission and neuronal output (Saraga et al., 2008; Wang and Maffei, 2014). This could reflect network’s requirement for a coordinated plasticity of inhibitory and excitatory inputs to maintain homeostasis, keep excitation/inhibition balance and prevent abnormal levels of activity.

One of the main components of LTP in the hippocampus is the increase in the efficacy of coupling between excitatory postsynaptic depolarization and spiking activity (Linden, 1999; Lu et al., 2000). Since perisomatic inhibition plays an essential role in determining the time window for spike generation (Pouille and Scanziani, 2001), increased inhibition through structural rearrangements of perisomatic synapses after plasticity inducing network activity may help in ensuring precise temporal synaptic integration. Addition or removal of GABAergic spine synapses in close proximity glutamatergic synapses in dendritic spines of cortical neurons allow the control of excitatory inputs through shunting inhibition (Maccaferri, 2005) and modulation of calcium signaling by near coincident activation of the GABAergic and excitatory inputs impinging on a particular spine (Chiu et al., 2013; Hayama et al., 2013) and it has the potential to affect processes of local, branch or dendritic segment specific computation (Pérez-Garci et al., 2006). In addition, dendrite inhibition can be very effective in damping neuronal activity by long-range shunting of excitatory inputs close to the soma. This means that plasticity of a few strategically located synapses could have important consequences for determining neuronal output (Gidon and Segev, 2012). In addition to its homeostatic role, inhibitory synapse structural plasticity may refine the synaptic basis of computational operations performed by hippocampal and cortical networks.

Experience shapes the formation and function of neuronal circuits during critical periods in early life (Berardi et al., 2000; Hensch, 2004). It seems increasingly clear that during these sensitive periods, special plasticity mechanisms that have a much smaller prevalence during adulthood are potentiated. In the visual cortex, where critical period has been extensively studied, the transition in and out of this critical period has been demonstrated to be under a strict control of the GABAergic system with a prominent role of perisomatic inhibition (Hensch, 2005). Changes in the rules of functional GABAergic synapse plasticity in cortical synapses that take place after the developmental switch in GABA synaptic signal polarity may play a prominent role in the transition into these periods of enhanced plasticity (Lefort et al., 2013). In addition, developmental and activity driven structural plasticity of GABAergic synapses may have important consequences for critical periods. In line with this, several studies have demonstrated that cortical sensory areas undergo an early disinhibition upon sensory deprivation (Micheva and Beaulieu, 1995; Keck et al., 2011; Chen et al., 2012; van Versendaal et al., 2012). It is likely that GABAergic synapse elimination is fundamental in allowing subsequent plastic changes in the cortex that may affect glutamatergic transmission. It is tempting to speculate that the interaction between GABAergic and glutamatergic synapses could involve a bidirectional crosstalk that include signals from the excitatory synapse that regulate GABAergic synapse strength and persistence (Marsden et al., 2010). Perineuronal nets (PNNs) are well organized structures formed by extracellular matrix molecules condensed around cell body and proximal dendrites of some types of neurons (Kwok et al., 2011). PNNs are formed by aggregation of heavily glycosilated proteins (proteoglycans) and, in the cortex, are preferentially associated with GABAergic cells (Morris and Henderson, 2000). Interestingly, PNNs are emerging as key structural regulators of INs plasticity (Wang and Fawcett, 2012). PNNs expression restricts neuronal plasticity by stabilizing synaptic connections and inhibiting activity dependent changes in neurotransmission (Berardi et al., 2004). Interestingly, disruption of PNNs con reactivate plasticity in some brain areas (Gogolla et al., 2009) and allows the reactivation of critical periods in the adult brain (Pizzorusso et al., 2002).

Changes in GABAergic transmission are essential for certain forms of memory and learning (Cui et al., 2008) but the information about the involvement of GABAergic synapse structural plasticity in network mechanisms of memory is still scarce. Some experiments have shown that learning-related behavior can drive long-lasting inhibitory synapse formation and elimination (Jasinska et al., 2010; Bloodgood et al., 2013) that may be responsible for durable changes in network connectivity underlying learning and memory. Behaviorally induced structural plasticity does not involve the general population of inhibitory synapses but differentially affects synapses impinging onto dendrites, spines or perisomatic compartment (Bloodgood et al., 2013). As a consequence of this differential regulation, experience may change the spread of inhibitory input among the different compartments and may affect information processing (Miles et al., 1996). Interestingly, selective deletion of Npas4 gene that codes for an activity regulated transcription factor responsible for differential remodeling of hippocampal dendritic and somatic GABA synapses in response to spatial exploration (Bloodgood et al., 2013), blocks hippocampal-dependent contextual learning (Ramamoorthi et al., 2011). Although Npas4 has been shown to control numerous genetic pathways that regulate both excitatory and inhibitory synapse function (Spiegel et al., 2014), its role in contextual memory formation could be at least in part mediated by an activity dependent remodeling of GABAergic synapses.

## Conclusions and future directions

In this review we have emphasized the role of synaptic activity in the remodeling of GABAergic synapse structure and discussed the possible roles of structural plasticity in sensory processing and memory formation. Although there is substantial experimental evidence of activity driven structural plasticity of GABAergic synapses, we have only partial knowledge of the implications of this type of plasticity for the function of inhibitory synapses and circuits and the molecular mechanisms that regulate different aspects of GABAergic synapse remodeling. In particular, several questions remain open for future investigation: (i) what are the structural determinants of GABAergic synapse function? (ii) what is the driving force for remodeling of GABA synapses, GABAergic or glutamatergic neurotransmission (or both)? (iii) how is the persistence of GABAergic synapses controled? (iv) how is structural plasticity of GABAergic and Glutamatergic synapses coordinated? (v) why spine inhibitory synapses are more dynamic than shaft inhibitory synapses? Addressing all these questions will surely advance our knowledge of the brain mechanisms of plasticity and define the precise roles of inhibitory synapse remodeling in the neuronal adaptation to experience, and in particular, for learning and memory.

## Conflict of interest statement

The authors declare that the research was conducted in the absence of any commercial or financial relationships that could be construed as a potential conflict of interest.
